# Selectivity Mechanism of the Voltage-gated Proton Channel, H_V_1

**DOI:** 10.1038/srep10320

**Published:** 2015-05-08

**Authors:** Todor Dudev, Boris Musset, Deri Morgan, Vladimir V. Cherny, Susan M. E. Smith, Karine Mazmanian, Thomas E. DeCoursey, Carmay Lim

**Affiliations:** 1 Institute of Biomedical Sciences, Academia Sinica, Taipei 115, Taiwan; 2 Faculty of Chemistry and Pharmacy, Sofia University, Sofia 1164, Bulgaria; 3 Institute of Complex Systems (ICS-4 Zelluläre Biophysik), Forschungszentrum Jülich, Jülich, NRW, Germany; 4 Department of Molecular Biophysics and Physiology, Rush University, Chicago, IL 60612, USA; 5 Department of Biology and Physics, Kennesaw State University, Kennesaw, GA 30144, USA; 6 Chemical Biology and Molecular Biophysics Program, Taiwan International Graduate Program, Academia Sinica, Nankang, Taipei 11529, Taiwan; 7 Department of Chemistry, National Tsing Hua University, Hsinchu 300, Taiwan

## Abstract

Voltage-gated proton channels, H_V_1, trigger bioluminescence in dinoflagellates, enable calcification in coccolithophores, and play multifarious roles in human health. Because the proton concentration is minuscule, exquisite selectivity for protons over other ions is critical to H_V_1 function. The selectivity of the open H_V_1 channel requires an aspartate near an arginine in the selectivity filter (SF), a narrow region that dictates proton selectivity, but the mechanism of proton selectivity is unknown. Here we use a reduced quantum model to elucidate how the Asp–Arg SF selects protons but excludes other ions. Attached to a ring scaffold, the Asp and Arg side chains formed bidentate hydrogen bonds that occlude the pore. Introducing H_3_O^+^ protonated the SF, breaking the Asp–Arg linkage and opening the conduction pathway, whereas Na^+^ or Cl^–^ was trapped by the SF residue of opposite charge, leaving the linkage intact, thus preventing permeation. An Asp–Lys SF behaved like the Asp–Arg one and was experimentally verified to be proton-selective, as predicted. Hence, interacting acidic and basic residues form favorable AspH^0^–H_2_O^0^–Arg^+^ interactions with hydronium but unfavorable Asp^–^–X^–^/X^+^–Arg^+^ interactions with anions/cations. This proposed mechanism may apply to other proton-selective molecules engaged in bioenergetics, homeostasis, and signaling.

The voltage-gated proton channel, H_V_1, has been implicated in numerous biological functions in humans^1^: charge compensation during the respiratory burst of phagocytes killing bacteria^2^,^3^, pH homeostasis in airway epithelia^4^, histamine secretion by basophils^5^, and triggering sperm capacitation^6^. It is a desirable and novel drug target^7^ due to its involvement in various inflammatory pathologies and its exacerbation of diseases such as ischemic stroke^8^, breast cancer^9^, and chronic lymphocytic leukemia^10^. In other species H_V_1 channels play diverse roles including mediating action potentials that trigger bioluminescence in dinoflagellates^11^ and enabling biogenic calcite production by coccolithophores as part of the global carbon cycle^12^. The ability of H_V_1 to perform its functions would fail if its proton selectivity were not perfect, due to the low concentration of protons in biological fluids. A conserved aspartate (Asp112 in humans) in the middle of the S1 transmembrane helix is an essential part of the H_V_1 selectivity filter (SF)^11^,^13^. This Asp consistently interacts with the second^14^,^15^ or third^16^,^17^ Arg in the S4 segment in homology models of human H_V_1 (hH_V_1) in an open (proton-conducting) conformation. However, it is seen to interact with the second Arg in the crystal structure of a closely related voltage-sensing phosphatase in the active conformation^18^. Classical molecular dynamics (MD) simulations indicate that charge compensation (e.g., an intact salt bridge) appears essential^19^, but do not reveal the mechanism by which proton selectivity occurs. Might selectivity result from obligatory protonation and deprotonation of a titratable group^13^,^20^ lining the SF? How does an Asp in a constricted SF select protons, while rejecting other cations/anions?

Takeshita *et al.*^21^ have determined a 3.45 Å structure of a chimeric murine H_V_1 channel in a probable closed conformation. This structure shows that the SF Asp is located in a hydrophobic layer comprising two conserved Phe residues, which might prevent water penetration. Presumably, this hydrophobic region prevents conduction of any ions including protons in closed channels. We adopt the nearly universal assumption that channel opening involves a protein conformational change. Opening allows H_3_O^+^ to access the SF from either side of the membrane. Since no 3D structure of H_V_1 in an open conformation has been solved, hypotheses on proton selectivity and conduction have been based on homology models derived from the open-state structures of voltage-gated sodium or potassium channels, which share only 13–19% sequence identity with hH_V_1^22^. MD simulations of hH_V_1 using as templates the open-state structures of the K_v_AP (1ORS)^23^ and the K_v_1.2-K_v_2.1 paddle chimera (2R9R)^16^ potassium channels predict a stable water wire in the open channel. It is widely accepted that protons can be conducted efficiently along a hydrogen-bonded water chain^24^,^25^,^26^,^27^,^28^. However, MD simulations of the same hH_V_1 channel derived from multiple templates (1ORS, 2R9R, and 3RVY)^14^ show that the Asp–Arg interaction, which interrupts the water wire, is only occasionally broken, yielding a transient water wire. Likewise, in simulations of *Ciona intestinalis* H_V_1^17^, which is homologous to hH_V_1 with 52% sequence identity, the average lifetime of a continuous water wire in an open-state model was only 6 ps. An ephemeral water-wire is suggestive of proton permeation involving titratable residues.

Whether proton selectivity could result from protonation/deprotonation of a titratable group can be answered only by considering explicit protonation/deprotonation reactions using all-electron quantum mechanical calculations, as done here. The lack of an open, proton-bound X-ray structure of hH_V_1 prohibits accurate evaluations of multi-ion free energy profiles for ion permeation. Thus, we evaluated selectivity by comparing the binding affinity of H_3_O^+^, Na^+^, Cl^–^, and H_2_O in the SF, assuming that the hH_V_1 would be selective to the permeating ion that binds with higher affinity in the SF. A reduced SF model was devised to capture the essential chemical processes underlying proton selectivity. It was designed to maximize resemblance to the open H_V_1 SF and was constructed on the basis of the following considerations: At the narrowest, relatively dry region of the pore^11^, the SF is lined by an aspartate (Asp112 in hH_V_1), which is conserved in all known and putative H_V_1^1^. This Asp interacts almost continuously with one of the three Arg residues in the S4 transmembrane segment in the open channel from MD simulations based on different homology models^14^,^15^,^16^,^17^,^29^. Even when the Asp was moved by double mutation from position 112 to 116 (D112V/V116D), it still interacted with one or two Arg residues with an intact or a broken salt-bridge in MD simulations^19^. Intriguingly, a positive point charge pulled through this double mutant in the broken configuration encountered a 10 kcal/mol barrier, but no barrier in the intact salt-bridge configuration^19^. These findings indicate that the Asp–Arg interaction is essential to proton selectivity, hence it was incorporated into the SF model. Ions such as Na^+^, OH^–^, and Cl^–^ were assumed to be dehydrated since the SF pore is purported to be narrow^14^,^21^. Ions in bulk solution were not included in the SF model, since H_V_1 channels are notoriously indifferent to ionic strength^13^, cations such as Ca^2+^ or Mg^2+^^30^,^31^, or anion species^31^.

To address whether proton selectivity arises from protonation and deprotonation of a titratable group, the interactions between the permeating ions and H_V_1 SF ligands, which play a key role in the competition between the native proton and its rivals, were treated explicitly using density functional theory to account for electronic effects such as polarization of the participating entities and differential amounts of ligand → ion charge transfer, while the region inside the SF was represented by a continuum dielectric. The proton was modeled as H_3_O^+^, while the Asp^–^, Arg^+^, Ala, His, and Lys^+^ side chains were modeled as -CH_2_-COO^–^, -CH_2_-NH-C(NH_2_)_2_^+^, -CH_2_-CH_3_, -CH_2_-imidazole, and -CH_2_-NH_3_^+^, respectively. The SF ligands were attached to a ring scaffold (see **Methods**), and the resulting complex was subject to all-electron geometry optimization without any constraints. The fully optimized SF geometries were then used to compute the ion-binding/exchange reactions in the H_V_1 pore characterized by an effective dielectric constant, *ε*. Since MD simulations of the open-state hHv1 model^14^ show that the SF is *not* in a bulk water environment but is relatively dry (see above), we employed *ε* ranging from 4 to 30^32^ to reflect a solvent-inaccessible or a partially solvent-exposed binding site, respectively, in order to encompass the actual value in the SF (see **Methods**). In interpreting results, we focus *not* on the absolute free energies, but on the change in ion-binding/exchange free energies with increasing *ε.* The approach outlined above has yielded structures and free energy trends in model SFs of various ion channels that are consistent with experimental findings^32^,^33^,^34^,^35^,^36^. The distance found here between the charge centers of the SF Asp and Arg (3.7 Å) agrees with that (3.8–4.6 Å) in MD simulations of the open hH_V_1^14^,^19^. The free energy trends in the model H_V_1 SF found herein are also consistent with experimental findings.

## Results

### Binding of H_3_O^+^ in the Asp–Arg SF

**Figure 1:**
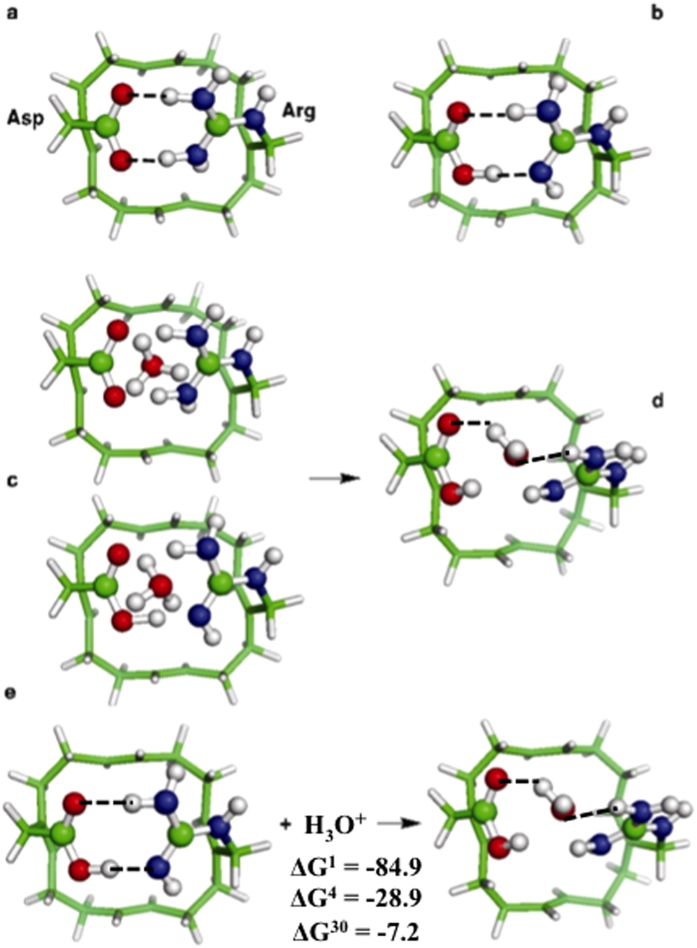
Binding of H_3_O^+^ to the Asp–Arg SF. Fully optimized B3-LYP/6-31+G(3d,p) structures of (a) ion-free Asp^–^–Arg^+^ SF, (b) Asp^0^–Arg^0^ SF, (c) initial configurations of the SF-H_3_O^+^ complex and (d) final configuration of the SF–H_3_O^+^ complex, AspH^0^–H_2_O–Arg^+^ with H in grey, C in green, N in blue and O in red. A dashed line denotes a hydrogen bond, which is defined by a donor–acceptor distance ≤3.5 Å and a H–acceptor distance ≤2.5 Å. The reaction between SF and H_3_O^+^ is depicted in (e) with free energies given in kcal/mol; ΔG^1^ is the binding free energy in the gas phase, whereas ΔG^4^ and ΔG^30^ are the corresponding free energies in the SF characterized by an effective dielectric constant of 4 and 30, respectively.

The ion-free Asp–Arg SF adopted two closed conformations that differ by <1 kcal/mol: an ion-pair conformation where the Asp and Arg side chains formed a bidentate salt bridge (Fig. 1a) and a hydrogen-bonded pair conformation where Arg protonated Asp, forming two hydrogen bonds (Fig. 1b). An Arg-carboxylate structural motif identified in several enzymes is thought to ensure rapid equilibrium between protonated and deprotonated Arg^37^. To see how the SF could accommodate passing ions, H_3_O^+^ was placed between Asp and Arg, above the hydrogen-bond network plane (Fig. 1c), mimicking the transient breaking of the Asp–Arg linkages, allowing H_3_O^+^ into the SF. The positioning of H_3_O^+^ between a deprotonated acid and a base has been observed spectroscopically^38^. In the final, fully optimized structure (Fig. 1d), the Asp and Arg side chains moved apart, breaking the two hydrogen bonds, thus opening the permeation pathway to accommodate the permeating H_3_O^+^, which transferred a proton to the SF leaving a water bridging AspH^0^ and Arg^+^. Binding of H_3_O^+^ to the Asp–Arg SF is thermodynamically favorable throughout the range of dielectric constant explored (negative ΔG^*x*^, Fig. 1e).

### Binding of Cl^–^ and Na^+^ to the Asp–Arg SF

**Figure 2:**
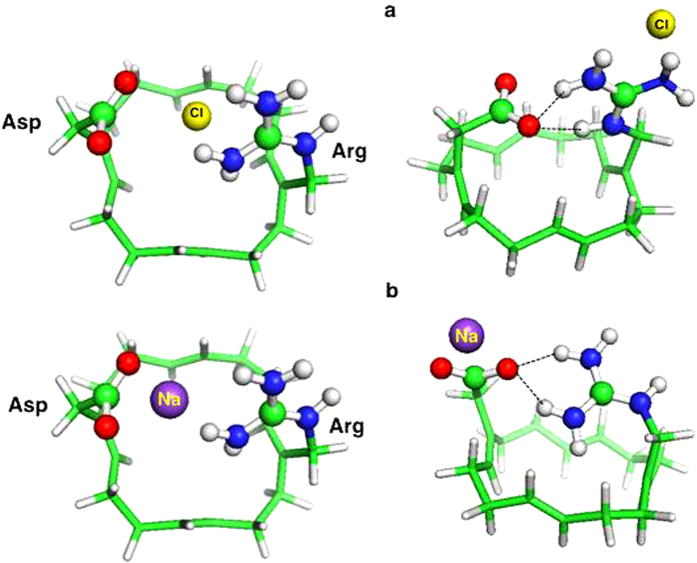
Binding of Cl^–^ and Na^+^ to Asp–Arg SF. Ball and stick diagrams of the initial (left) and final (right) structures of SF complexes with (a) Cl^–^ and (b) Na^+^.

The Asp–Arg SF responded quite differently to the introduction of the proton’s competitors, Cl^–^ and Na^+^. We started from the “open” pore structure, where the Asp and Arg side chains were separated, and placed the incoming ion between them (Fig. 2, left). Such a configuration was not favorable as during geometry optimization, the introduced ion was ejected from the pore, away from the residue bearing the same charge and became trapped by the residue carrying the opposite charge: Arg^+^ for Cl^–^ and Asp^–^ for Na^+^ (Fig. 2, right). In contrast to the open starting structures, the hydrogen-bond network between Asp and Arg was partially restored in the final optimized structures, closing the SF aperture and excluding other ions.

The above results highlight the importance for proton selectivity of electrostatic interactions between the SF and permeating ions. The SF Asp–Arg pair intrinsically selects protons and rejects other cations and anions: the only species that can bind favorably to both Asp^–^ and Arg^+^ in an “open” state is H_3_O^+^ (Fig. 1e). Cl^–^ and Na^+^ are not permeable, as they do not promote pore opening (Fig. 2).

### H_2_O vs. H_3_O^+^ Binding in the Asp–Arg SF

**Figure 3:**
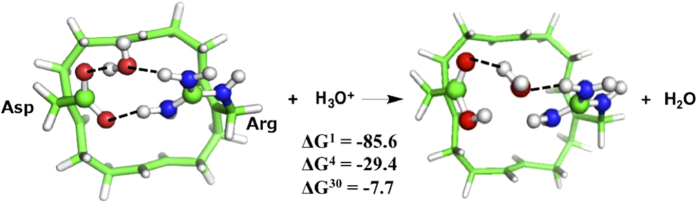
Free energies (in kcal/mol) for replacing H_2_O bound in Asp–Arg SF with H_3_O^+^. See Fig. 1 legend.

Although the Asp112–Arg208 pair is broken only 10% of the time in MD simulations of a homology model of hH_V_1 in an open conformation, this transient disruption allows formation of a water wire that could last for 1 ns^14^. Would a water molecule be even more stable than H_3_O^+^ in the H_V_1 SF? In other words, can H_3_O^+^ displace water bound to the Asp–Arg pair? To address this question, we placed H_2_O in between the Asp–Arg pair and optimized the structure. The fully optimized structure in Fig. 3 (left) shows that a water molecule, unlike H_3_O^+^, cannot fully dissociate the Asp–Arg pair, as a hydrogen bond remains between the two residues. Furthermore, H_3_O^+^ can easily displace water bound to the Asp–Arg pair and protonate Asp (Fig. 3, right): The computed free energies (ΔG^*x*^, *x* = 1–30) for H_3_O^+^ to displace H_2_O from the Asp–Arg pair are all favorable (negative ΔG^*x*^, Fig. 3). The positive free energies for the reverse reaction imply that a water molecule cannot readily displace H_3_O^+^ bound to the Asp–Arg pair.

### The Arg208Lys Mutant is Predicted to be Proton-selective

**Figure 4:**
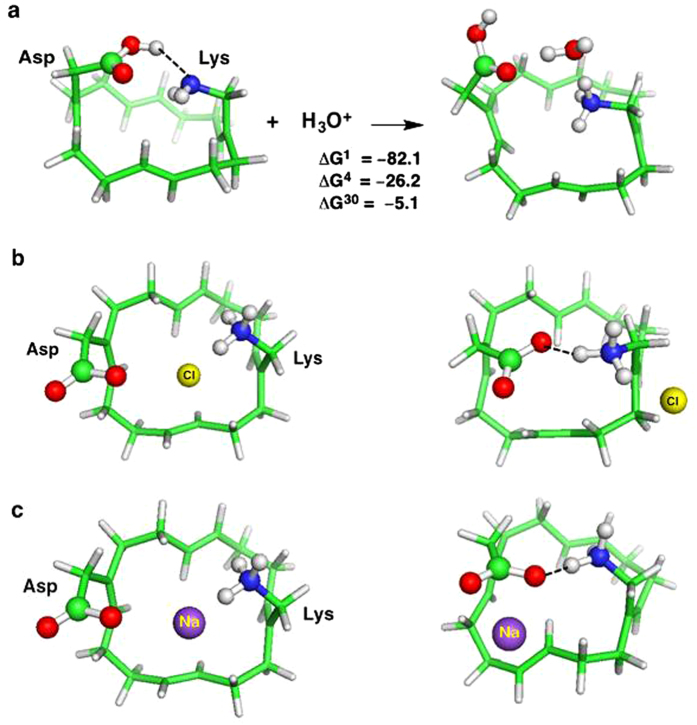
(a) Free energies (in kcal/mol) for binding of H_3_O^+^ to Lys mutant SF. Ball and stick diagrams of the initial (left) and final (right) structures of Arg → Lys mutant SF complexes with Cl^–^ (b) and Na^+^ (c). See Fig. 1 legend.

Replacing the Lys lining the pore of voltage-gated Na^+^ channels with Arg nearly abolishes the channel’s selectivity for Na^+^ over K^+^^39^. Is Arg in the H_V_1 SF likewise indispensable for proton selectivity? To address this question, we replaced the SF Arg by Lys and evaluated its proton selectivity. Lys behaved like its Arg counterpart: in the ion-free state, Lys protonated Asp forming a hydrogen bond (Fig. 4a, left); however, because Lys has a lower pK_a_ than Arg, a stable Asp^–^–Lys^+^ ion pair minimum could not be found. In the ion-bound state, H_3_O^+^, which was initially placed between the protonated Asp and neutral Lys, transferred a proton to the SF leaving a water molecule to bridge AspH^0^ and Lys^+^ (Fig. 4a, right). The AspH^0^–H_2_O–Lys^+^ complex formation free energies remain thermodynamically favorable, although slightly less so than those for the wild-type Asp–Arg SF (compare numbers in Figs. 1e and 4a). As in the wild-type SF, during geometry optimization, Cl^–^ and Na^+^ were repelled by the SF residue of the same net charge and moved towards the SF residue with the opposite charge. In the final optimized structures, Asp^–^ and Lys^+^ formed a hydrogen bond, prohibiting the competing Cl^–^ and Na^+^ ions from passing through the pore (Figs. 4b and 4c).

**Figure 5:**
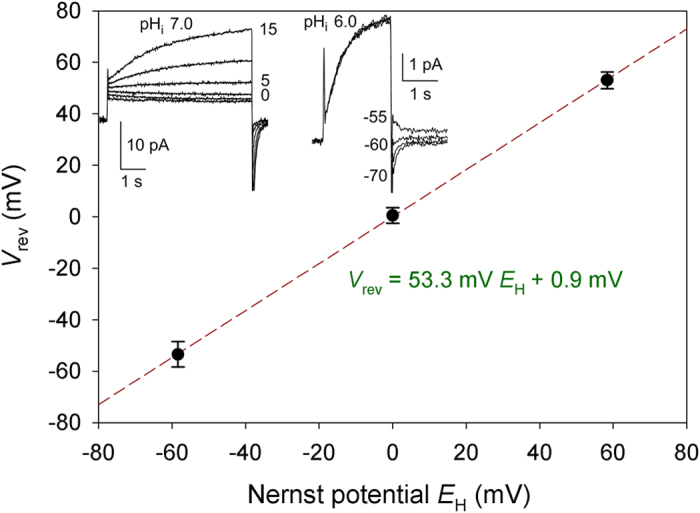
The Lys208 mutant is proton selective. Measured values of *V*_rev_ at ΔpH –1.0, 0, or 1.0 (mean ± SEM, *n* = 3, 9, or 6, respectively), with pH_o_ ranging 5.5 to 7.0 and pH_i_ ranging 5.5 to 8.0. The linear regression slope was 53.3 mV/unit ΔpH, compared with the Nernst value of 58.4 mV. *Inset*: Proton currents in an inside out patch during pulses applied in 5 mV increments (left) indicate reversal between 0 and 5 mV (the conductance activated negative to *V*_rev_) at pH_i_ 7.0, with pH_o_ 7.0 (in the pipette). Tail currents in the same patch at pH_i_ 6.0 indicate reversal at –58 mV. Both values are near the Nernst predictions of 0 mV and –58.4 mV.

The prediction that the Lys mutant SF is selective for protons over other competing ions was verified experimentally by mutating Arg208 lining the SF to Lys: currents through the Lys208 mutant reversed near the Nernst potential for H^+^ (Fig. 5); the reversal potential (*V*_rev_) did not change when Na^+^ or K^+^ replaced TMA^+^ or Cl^–^ replaced CH_3_SO_3_^–^ (Supplementary Table S5).

### Why D112A and D112H Mutants are Chloride-selective

**Figure 6:**
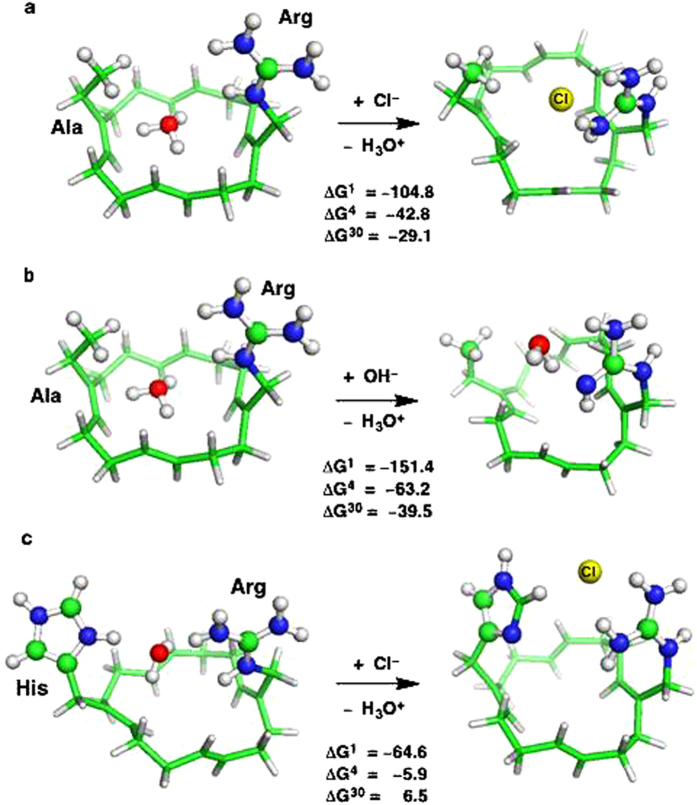
Binding of Cl^–^ and/or OH^–^ to H_3_O^+^-bound mutant SFs. B3LYP/6-31+G(3d,p) fully optimized structures of H_3_O^+^–SF, Cl^–^–SF and OH^–^–SF complexes, and free energies (in kcal/mol) for (a) [SF(Ala-Arg^+^)-H_3_O^+^] + Cl^–^ → [SF(Ala-Arg^+^)-Cl^–^] + H_3_O^+^, (b) [SF(Ala-Arg^+^)-H_3_O^+^] + OH^–^ → [SF(Ala-Arg^+^)-OH^–^] + H_3_O^+^, and (c) [SF(His-Arg^+^)-H_3_O^+^] + Cl^–^ → [SF(His-Arg^+^)-Cl^–^] + H_3_O^+^. ΔG^1^ is the ion exchange free energy in the gas phase, whereas ΔG^4^ and ΔG^30^ are the corresponding free energies in the SF characterized by an effective dielectric constant of 4 and 30, respectively. If the resulting free energy is negative, the pore is Cl^–^ or OH^–^-selective, but if it is positive, the pore is proton-selective.

Mutagenesis studies^13^ show that replacing Asp112 in the SF with a neutral residue such as Ala or the weak base His converts the channel into an anion-selective pore. Why? To address this question we modeled two types of SF mutants: Ala^0^–Arg^+^ (Fig. 6a,b) and His^0^–Arg^+^ (Fig. 6c). Replacing anionic Asp112^–^ with neutral Ala or His leaves the positive charge on the SF Arg^+^ uncompensated, which disfavors H_3_O^+^ binding to the SF due to the like charge repulsion between H_3_O^+^ and Arg^+^. On the other hand, strong attractive forces between the permeating OH^–^/Cl^–^ and Arg^+^ stabilize the OH^–^/Cl^–^–SF complexes, and thus favor binding of the anion. To verify that the Ala112 and His112 mutants would be anion-selective, we computed the free energy for replacing H_3_O^+^ in the mutant SFs with Cl^–^. In line with the experimental observations, the Ala^0^–Arg^+^ SF is highly Cl^–^-selective in both solvent-inaccessible and exposed pores (negative Δ*G*^*x*^, Fig. 6a). It is predicted to be even more selective for OH^–^ (more negative Δ*G*^*x*^ in Fig. 6b than in Fig. 6a), in accord with the experimental finding that the Asp112Ala mutant is more permeable to OH^–^ than to Cl^–13^. This is likely so because the SF Arg can protonate OH^–^, yielding a neutral Ala^0^–H_2_O^0^–Arg^0^ complex.

Like the Ala^0^–Arg^+^ mutant, the His^0^–Arg^+^ SF is predicted to be also anion-selective provided the narrow pore has limited solvent accessibility (negative ΔG^4^), which is seen in the 3.45 Å crystal structure of a mouse H_V_1 chimeric channel (PDB 3WKV)^21^ and in simulations of open-state H_V_1 models^14^,^17^. However, it is predicted to be less Cl^–^-selective than the Ala^0^–Arg^+^ filter (less negative ΔG^4^ in Fig. 6c than in Fig. 6a), which is also consistent with experiment^13^. This is largely because H_3_O^+^ protonated the His–Arg SF, stabilizing the His^+^–H_2_O–Arg^+^ “reactant” complex (Fig. 6c, left), but no such stabilization can occur in the Ala^0^–H_3_O^+^–Arg^+^ “reactant” complex (Fig. 6a, left).

## Discussion

Previous studies^16^,^23^ have proposed that a water wire might conduct protons through H_V_1, but this does not explain how other ions are excluded and why an aspartate (Asp112 in humans) in the H_V_1 pore is essential for proton selectivity^11^,^13^. This work shows that the H_V_1 Asp–Arg SF selects protons by transferring a proton from H_3_O^+^ to the SF, highlighting the importance of quantum effects (charge transfer and polarization). Although a water molecule can be inserted between Asp and Arg, it is readily displaced by H_3_O^+^ (Fig. 3), which then transfers its extra proton to the SF.

**Figure 7:**
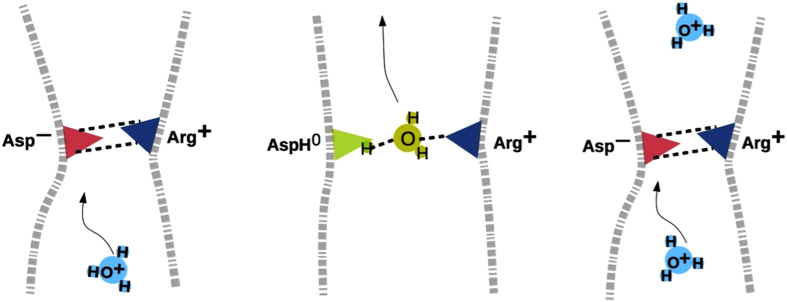
Schematic cartoon of the proposed proton selectivity mechanism by the H_V_1 SF. Negatively charged Asp is red, neutral AspH^0^ and H_2_O^0^ are green, whereas positively charged H_3_O^+^ and Arg are light and dark blue, respectively. The dashed lines denote hydrogen bonds or salt bridges that occlude the SF pore. When H_3_O^+^ approaches the SF (left), it breaks the hydrogen bonds and protonates the SF, resulting in neutral H_2_O bridging AspH^0^ and Arg^+^ (middle). Transfer of a proton from the SF to H_2_O completes the conduction cycle (right).

This work suggests the following proton selectivity mechanism in the H_V_1 SF: On a time-scale of seconds, the channel helices, S4 in particular^18^,^40^, move from a closed conformation that does not allow conduction to an open one that does. For other ion channels, opening produces a continuous water-filled pore, through which water and ions pass, often in single-file through the narrowest region^41^,^42^. For H_V_1, channel opening produces instead a relatively dry pore that is constricted by two hydrogen bonds formed by the SF Asp and Arg^14^ (Fig. 1a,b). Thermal fluctuations could transiently break the Asp–Arg linkage, allowing ions or water to approach the narrow SF (Fig. 1c, Figs. 2 and 3, left). The permeating H_3_O^+^ protonates the SF Asp, resulting in favorable AspH^0^–H_2_O^0^–Arg^+^ interactions (Fig. 1d), thus “opening” the pore to enable its own permeation, whereas anions (X^–^) or cations (X^+^) encounter unfavorable Asp^–^–X^–^–Arg^+^ or Asp^–^–X^+^–Arg^+^ interactions, and are ejected, restoring the Asp-Arg linkage (Fig. 2, right). Hence, the H_V_1 Asp–Arg SF intrinsically selects protons by virtue of its ability to “close” its pore when H_3_O^+^ is absent, to “open” its pore by accepting a proton when H_3_O^+^ enters, while rejecting other cations and anions though electrostatic repulsion. In the absence of permeating ions, the SF residues form hydrogen bonds that occlude the pore. Among cations, H_3_O^+^ is uniquely able to protonate the SF ligands, permeate as neutral H_2_O, and then retrieve the excess proton (Fig. 7).

**Figure 8:**
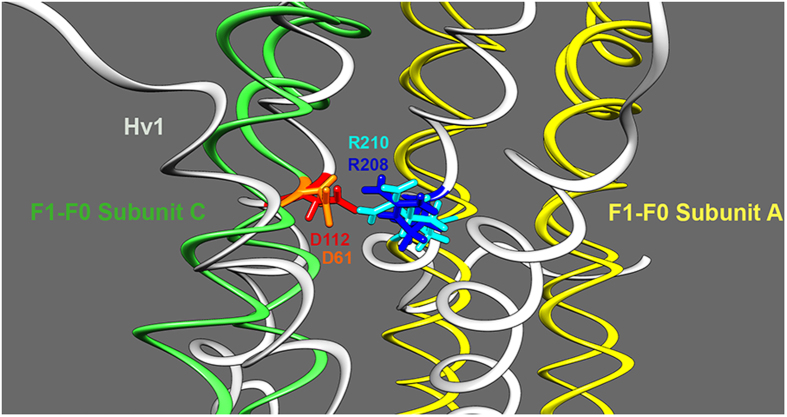
A critical Asp-Arg pair in F_1_-F_o_ ATPase shares similar geometry to that in H_V_1. Based on a homology model of H_V_1 in the open state^14^ and the crystal structure of F_1_-F_o_ ATPase (PDB ID 1C17), Asp112 in H_V_1 was superimposed onto Asp61 of F_1_-F_o_ subunit *c* using Chimera, which minimizes the root-mean-square deviations of superimposed atoms. This resulted in Arg208 of H_V_1 occupying a similar position to Arg210 of F_1_-F_o_ subunit *a*, which is known to participate in proton translocation.

The mechanism for proton selectivity found herein may also apply to other molecules. For example, if Asp112 from human H_V_1 is superimposed on Asp61 of the F_o_F_1_-type H^+^-ATPase, Arg210 aligns with Arg208 of H_V_1 (Fig. 8). Asp61 and Arg210 are located in the proton pathway of this H^+^-ATPase and are the only two amino acids that are absolutely required for function^43^.

Several other proteins, which have Asp–Arg/Lys pairs thought to be critical to proton transport, also exhibit distances between the charge centers similar to the pair in H_V_1. Examples of such proteins and the distances between charge centers include Na^+^ phosphatase, 3.9 Å^44^; H^+^ phosphatase, 4.0 Å^45^; and the glucose H^+^ symporter XylE, 4.1 Å^46^. In the Asp–Arg motif common to several proton pumps, a function of Arg is thought to be electrostatic ejection of the proton at the appropriate moment in the pump cycle^43^,^47^. This interacting charge pair may help enforce proton selectivity in these molecules, as in H_V_1.

Conversely, we searched for Asp–Arg pairs in pores of non-proton channels, where such linked acid-base pairs should not exist. We examined 60 ion channels and transporters (including various cation and anion channels, aquaporin, and organic cation transporters) for which X-ray structures exist (see Supplementary Table S6). Following criteria for a proton SF established previously^19^, we searched for a pore-facing Asp/Glu in hydrogen-bond contact with a single Arg/Lys, located in a narrow region of the pore in an open conformation. We found no counterexample contradicting our hypothesis.

Although the interactions between ions and the known SF ligands (notably, both amino acids directly implicated in selectivity by mutation studies) have been treated in detail using all-electron quantum mechanical calculations, the contributions from other segments of the pore and ions have not been modeled explicitly in the absence of a high-resolution structure of the open-state H_V_1 channel. Consequently, the present results, which are in line with experimental observations, are limited to explaining proton selectivity in the constricted, relatively dry Asp-Arg SF. How the proton leaves this SF is not explicitly dealt with here. Perhaps an incoming H_3_O^+^ (or another cation) could dislodge H_3_O^+^ from the SF, as in the classical “knock-on” mechanism for K^+^ channels proposed by Hodgkin and Keynes^42^. MD simulations of the open hH_V_1 channel derived from multiple templates^14^ show that the SF is located at the extracellular end of a narrow constriction ~10 Å long with a hydrophobic region surrounding Phe150–Arg211^14^,^15^ at the inner end. Thus, another question is how protons pass through this second Phe150-Arg211 hydrophobic zone. However, in a recent computational study^48^, H_3_O^+^ positioned at the entrance to a hydrophobic pore was found to induce water entry, creating its own water wire and lowering the free energy barrier for proton permeation. Such a mechanism may transiently hydrate the Phe-Arg bottleneck, enabling proton hopping from one water molecule to the next. When the open H_V_1 channel structure becomes available, the contributions of non-SF residues, proton coupling, and kinetic barriers to proton selectivity could be assessed from computed charge-transfer free energy profiles.

## Methods

### SF Model and Justification

Models of the hH_V_1 SFs were built using GaussView version 3.09, following the guidelines from our previous work^32^. The SF ligating groups were coordinated to the permeating ion or water and attached to a carbon–hydrogen ring scaffold via flexible methylene spacers (see Figures). The ring scaffold prevents the metal ligands from drifting away or assuming unrealistic, pore-occluding positions during geometry optimization. However, the shape and the C–H orientations of the ring do not obstruct the pore lumen. Moreover, the ion-ligating groups and their connection to the ring are flexible enough to allow them to optimize their positions upon ion/water binding.

### Geometry Optimization of the SF Model

In previous studies^32^, the B3-LYP/6-31+G(3d,p) method was shown to be the most efficient among the various methods tested in reproducing experimentally determined molecular properties and structural characteristics of model ligands and metal complexes (see Supplementary Table S1). Hence, it was used to optimize the geometry of each model SF without any constraints and to compute the electronic energies, *E*_*el*_, using the Gaussian 09 program. It was also used to compute the frequencies of each optimized structure. No imaginary frequency was found in any of the optimized structures.

### Free Energy Calculations

The binding of H_3_O^+^ to a model SF to yield [H_3_O^+^-SF] is described by the following reaction




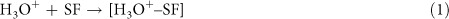




Binding of H_3_O^+^ to the wild-type or mutant H_V_1 SF is thermodynamically favorable only if the binding free energy for eq 1 is negative. Following Eisenman’s equilibrium theory of ion selectivity^49^, the filter’s selectivity can be expressed in terms of the free energy Δ*G*^*x*^ for replacing the native H_3_O^+^ bound inside a model SF, [H_3_O^+^–SF], with a rival ligand such as water, Na^+^, Cl^–^ or OH^–^ (denoted as X)




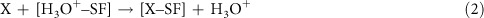




The native H_3_O^+^ is preferred to the rival ligand X in the wild-type or mutant H_V_1 SF if Δ*G*^*x*^ for eq 2 is positive or if Δ*G*^*x*^ for the reverse reaction, [X–SF] + H_3_O^+^ → X + [H_3_O^+^–SF], is negative. Na^+^ or Cl^–^ in the SF was unstable and was found near the side chain of opposite charge in the final optimized structures, precluding determination of its binding affinity.

The reaction in eq 1 or 2 was modeled to occur in vicinity of the SF so that the dielectric environment *ε* was assumed to be uniform for all participating entities; the respective free energy was computed using the following thermodynamic cycle:




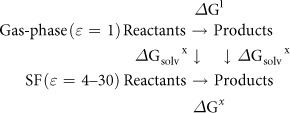




Thus, the free energy for eq 1 or 2 can be computed as a sum of the gas-phase free energy ΔG^1^ and the solvation free energy ΔΔG_solv_^x^ difference between the products and reactants; i.e.,




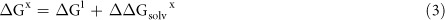




The gas-phase free energy, ΔG^1^, was computed from the electronic energy (ΔE_el_), thermal energy (ΔE_th_), work term (ΔPV), and entropy differences between products and reactants,




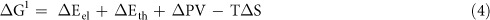




The thermal energies including zero-point energy and entropies were computed from the B3-LYP/6-31+G(3d,p) frequencies scaled by an empirical factor of 0.9613^50^.

The solvation free energy, ΔG_solv_^x^, was estimated by solving Poisson’s equation with the MEAD program^51^ using natural bond orbital atomic charges^52^ and the following effective solute radii (in Å): *R*_H_ = 1.50, *R*_H_(H_3_O^+^) = 1.05, *R*_Na_ = 1.72, *R*_C_ = 1.95, *R*_N_ = 1.75, *R*_O_(H_2_O) = 1.85, *R*_O_(H_3_O^+^) = 1.65, *R*_O_(HO^–^) = 1.64, *R*_O_(COO^–^) = 1.56, and *R*_Cl_ = 2.30. The computed hydration free energies of the cations and ligands could reproduce the experimental values^32^,^34^,^53^ (Supplementary Table S2).

### Validation against Experimental Free Energies

The methodology used to compute Δ*G*^*x*^ has been validated against experimental ion exchange free energies between biogenic metal cations (Na^+^, K^+^, and Ca^2+^) in crown ethers, which resemble SF pores^32^, and in systems containing carboxylic ligands (nitrilotriacetic acid)^34^. The computed metal exchange free energies can reproduce the corresponding experimental values to within 1 kcal/mol (Supplementary Table S3)^32^,^34^,^53^. The methodology has yielded trends in the free energy changes that are in accord with experimental findings^32^,^33^,^34^,^35^,^36^,^53^,^54^,^55^,^56^. It has also yielded calculated pore aperture areas in good agreement with experimental estimates (Supplementary Table S4).

## Author Contributions

T.D. performed the calculations. B.M., D.M., and V.C. conducted patch-clamp studies and analyzed results. S.M.E.S. provided constructs. S.M.E.S. and K.M. performed PDB data analysis. T.D., S.M.E.S. and K.M. prepared figures, T.E.D. and C.L. designed the project and discussed results. T.D., T.E.D., and C.L. participated in writing the manuscript.

## Additional Information

**How to cite this article**: Dudev, T. *et al*. Selectivity Mechanism of the Voltage-gated Proton Channel, H_V_1. *Sci. Rep.* **5**, 10320; doi: 10.1038/srep10320 (2015).

## Supplementary Material

Supplementary Information
