# Supplementary material for: Selectivity Mechanism of the Voltage-gated Proton Channel, HV1
Source: Sci Rep. 2015 May 8;5:10320. doi: 10.1038/srep10320 (PMC4429351; doi:10.1038/srep10320)
Supplement: Supplementary Information [file srep10320-s1.pdf]

Selectivity Mechanism of the Voltage-gated Proton Channel, H_V1

Todor Dudev[1,2*], Boris Musset[3], Deri Morgan[4], Vladimir V. Cherny[4], Susan M.E. Smith[5], Karine Mazmanian,[1,6] Thomas E. DeCoursey[4*], and Carmay Lim[1,7*]

[1]Institute of Biomedical Sciences, Academia Sinica, Taipei 115, Taiwan, [2]Faculty of Chemistry and Pharmacy, Sofia University, Sofia 1164, Bulgaria, [3]Institute of Complex Systems (ICS-4 Zelluläre Biophysik), Forschungszentrum Jülich, Jülich, NRW, Germany, [4]Department of Molecular Biophysics and Physiology, Rush University, Chicago, IL 60612, USA, [5]Department of Biology and Physics, Kennesaw State University, Kennesaw, GA 30144, USA, [6]Chemical Biology and Molecular Biophysics Program, Taiwan International Graduate Program, Academia Sinica, Nankang, Taipei 11529, Taiwan, [7]Department of Chemistry, National Tsing Hua University, Hsinchu 300, Taiwan

Supplementary Table S1. Calculated and experimental molecular dipole moments of water, methanol and formaldehyde (in Debye).

Method	H_2O	CH_3OH	$HCONH_2$
HF/6-31+G(d,p)	2.23	1.97	4.30
HF/6-31+G(2d,2p)	2.03	1.80	4.19
HF/6-31+G(3d,p)	1.96	1.74	4.15
HF/6-31+G(3d,2p)	1.97	1.75	4.14
HF/6-311++G(d,p)	2.20	1.94	4.22
HF/6-311++G(3df,3pd)	1.97	1.74	4.12
MP2/6-31+G(d,p)	2.28	2.07	4.50
MP2/6-31+G(2d,2p)	2.08	1.89	4.38
MP2/6-31+G(3d,p)	2.01	1.83	4.34
MP2/6-31+G(3d,2p)	2.02	1.83	4.33
MP2/6-311++G(d,p)	2.26	2.03	4.38
MP2/6-311++G(3df,3pd)	2.02	1.82	4.29
SVWN/6-31+G(d,p)	2.25	1.86	4.11
SVWN/6-31+G(2d,2p)	2.01	1.68	4.03
SVWN/6-31+G(3d,p)	1.90	1.61	3.99
SVWN/6-31+G(3d,2p)	1.91	1.62	3.98
SVWN/6-311++G(d,p)	2.21	1.84	4.05
SVWN/6-311++G(3df,3pd)	1.91	1.61	3.95
B3LYP/6-31+G(d,p)	2.19	1.91	4.11
B3LYP/6-31+G(2d,2p)	1.98	1.73	4.03
**B3LYP/6-31+G(3d,p)**	**1.88**	**1.67**	**3.99**
B3LYP/6-31+G(3d,2p)	1.89	1.68	3.98
B3LYP/6-311++G(d,p)	2.16	1.89	4.05
B3LYP/6-311++G(3df,3pd)	1.89	1.67	3.96
Experiment from Lide, 2006.[1]	1.85 ± 0.02	1.70 ± 0.02	3.73 ± 0.07

Supplementary Table S2. Comparison between Computed and Experimental Hydration Free Energies, ΔGsolv80, of Metal Cations and Ligands (in kcal/mol).

Metal/Ligand	ΔG_{solv}^{80}		
	Expt	Calcd	Error[a]
Na^+	–98.3[b]	–98.7	–0.4
K^+	–80.8[b]	–81.0[c]	–0.2[c]
		–80.9[d]	–0.1[d]
		–81.2[e]	–0.4[e]
Ca^{2+}	–380.8[b]	–381.1	–0.3
H_2O	–6.3[f]	–6.7	–0.4
CH_3OH	–5.1[g]	–6.1	–1.0
$HCONH_2$	–10.0[h]	–10.6	–0.6
CH_3COO^-	–82.2[i]	–82.3	–0.1

[a]Error = ΔG_{solv}^{80}(Calcd) - ΔG_{solv}^{80}(Expt). [b]From Friedman & Krishnan, 1973.[2] [c]Hexahydrated K^+. [d]Heptahydrated K^+. [e]Octahydrated K^+. [f]From Ben-Naim & Marcus, 1984.[3] [g]From Chambers et al., 1996.[4] [h]Experimental solvation free energy of $HCONH(CH_3)$ from Wolfenden, 1978.[5] [i]From Lim et al., 1991.[6]

Supplementary Table S3. Comparison between Computed and Experimental Free Energies of Metal Exchange, ΔG_{ex}^{80}, in 18-crown-6 and Nitrilotriacetic Acid (NTA) Complexes (in kcal/mol).

Reaction	ΔG_{ex}^{80} (kcal/mol)		
	Expt	Calcd	Error[a]
$[Na(H_2O)_6]^+$ + [K(18-crown-6)]$^+$ → $[K(H_2O)_6]^+$ + [Na(18-crown-6)]$^+$	2.0[b]	1.4	–0.6
$[Na(H_2O)_6]^+$ + H_2O + $[Ca(H_2O)_2(NTA)]^-$ → $[Na(H_2O)_2(NTA)]^{2-}$ + $[Ca(H_2O)_7]^{2+}$	7.1[c]	6.7	–0.4

[a]Error = ΔG_{solv}^{80}(Calcd) - ΔG_{solv}^{80}(Expt). [b]From Ozutsumi & Ishigiro, 1992.[7] [c]Calculated from the experimental stability constants of the respective metal complexes from Smith & Martell, 1987.[8] NTA binds in a tetradentate fashion (including central N atom) to the metal.

Supplementary Table S4. Comparison between computed and experimentally determined areas of various sodium channel SF pores.

Na Channel	SF type	Area (Calc)[a] $Å^2$	Area (Expt)[a] $Å^2$
Epithelial	Na-BBB/SSS	6.3/6.9[b]	<8.1[c]
Eukaryotic Na_v	Na-DEKA	5.4[d]	4.2[e]
Acid-sensing	Na-GGG-3/6	15.7[b]	16.6[f]
Bacterial Na_v	Na-2E+2W	22	21[g]

[a]Calculated as the area of the triangle or quadrangle (for Na-2E+2W) formed by the metal ligating oxygen atoms lining the SF; see Figure 3 in Dudev & Lim, 2012.[9]
[b]From Dudev & Lim, 2015.[10]
[c]From Kellenberger et al., 1999[11] where pore diameter is <5.0 Å.
[d]From Dudev & Lim, 2010.[12]
[e]From Sun et al., 1997[13] where pore diameter is 3.6 Å.
[f]From Baconguis et al., 2014.[14]
[g]From Payandeh et al., 2011[15] where pore diameter is 3.2 Å.

Supplementary Table S5. Reversal potentials of currents through the H_V1 K^{208} mutant.[a]

Ion	ΔV_{rev} (mV)
Na^+	1.2 ± 1.3 (3)
K^+	−1.9 ± 2.0 (4)
Cl^-	2.9 ± 1.6 (5)

[a]The change in V_{rev} when the specified ion replaced TMA^+ or $CH_3SO_3^-$ is given. Values are corrected for the liquid junction potential measured in each solution, and include measurements at symmetrical pH 5.5 or 7.0. The changes are smaller than the liquid junction potential correction and than the variability of the measurements themselves. No other ion was detectably permeant.

Supplementary Table S6. PDB List Searched for Asp-Arg pairs

PDB ID	Name	Selectivity
**1K4C**	KcsA Potassium channel, H+ gated (high K+ concentration)	**K+ channel**
**1K4D**	KcsA Potassium channel, H+ gated (low K+ concentration)	**K+ channel**
**1OTS**	H(+)/Cl(-) exchange transporter ClcA	**Cl-/H+ exchange transporter**
**1YMG**	Lens fiber major intrinsic protein	**water channel**
**2A0L**	Voltage-gated potassium channel KvAP	**K+ channel**
**2A79**	Shaker Kv1.2 Kv1.2/Kv2.1 Voltage-gated potassium channel chimera	**K+ channel**
**2ABM**	Aquaporin Z	**water channel**
**2BG9**	Acetylcholine receptor subunit alpha (closed state)	**cation channel**
**2NUU**	Ammonia channel AmtB	**ammonia channel**
**2OAR**	Large-conductance mechanosensitive channel MscL	**ion channel**
**2OAU**	Small-conductance mechanosensitive channel MscS	**ion channel**
**2VV5**	Small-conductance mechanosensitive channel MscS (open structure)	**ion channel**
**2X6A**	Inward rectifier potassium channel Kirbac3.1 (semi-latched)	**K+ channel**
**2ZD9**	Cyclic nucleotide-gated potassium channel mll3241 MlotiK1	**K+ channel**
**3B9W**	Ammonium transporter family Rh50	**ammonia channel**
**3C1H**	Ammonia channel AmtB	**ammonia channel**
**3E83**	NaK channel	**K+ Na+ channel**
**3HZQ**	Large-conductance mechanosensitive channel MscL (expanded intermediate state)	**ion channel**
**3K07**	Cation efflux system protein CusA	**Cu+ and Ag+ channel**
**3LUT**	Shaker Kv1.2 Kv1.2/Kv2.1 Voltage-gated potassium channel chimera (full length)	**K+ channel**
**3M71**	Tellurite resistance protein TehA homolog	**anion channel**
**3PJS**	pH-gated potassium channel KcsA (full length)	**K+ channel**
**3RHW**	Glutamate-gated chloride channel alpha	**chloride channel**
**3RQU**	Cys-loop ligand-gated ion channel ELIC	**cation channel**
**3RVY**	Voltage-Gated Sodium Channel (NaV)	**Na+ channel**
**3S3W**	Acid-Sensing Ion Channel 1 ASIC1 (pH 7.5)	**Na+ channel**
**3SPC**	ATP-sensitive inward rectifier potassium channel 12 Kir2.2 (Complete)	**K+ channel**

PDB ID	Name	Selectivity
**3SYC**	GIRK2 (Kir3.2) G-protein-gated K+ channel	**K+ channel**
**3UKM**	Potassium channel subfamily K member 1	**K+ channel**
**3UM7**	Potassium channel subfamily K member 4	**K+ channel**
**3ZJZ**	NavMs channel from Magnetococcus marinus (open state)	**Na+ channel**
**3ZKR**	Cys-loop ligand-gated ion channel ELIC	**cation channel**
**3ZOJ**	Aquaporin PIP2-7 7 Aqy1	**water channel**
**3ZRS**	KirBac3.1 ATP-sensitive Inward-Rectifier Potassium channel 10 (semi-latched)	**K+ channel**
**4DW0**	ATP-gated P2X4 ion channel (closed, apo state)	**cation channel**
**4DXW**	Na(v)Rh Voltage-Gated Sodium Channel	**Na+ channel**
**4EED**	Magnesium transport protein CorA	**Mg2+ channel**
**4F4L**	NavMs Voltage-Gated Sodium Channel (apo structure)	**Na+ channel**
**4GX5**	GsuK multi-ligand gated K+ channel	**K+ channel**
**4H33**	KvLm voltaged-gated potassium channel	**K+ channel**
**4HKR**	Calcium release-activated calcium channel protein 1 (CRAC)	**Ca2+ channel**
**4HYO**	Calcium-gated potassium channel MthK	**K+ channel**
**4I9W**	Potassium channel subfamily K member 4 (K2P4.1)	**K+ channel**
**4K7R**	Cation efflux system protein CusC	**Cu+ and Ag+ channel**
**4LMJ**	Proton-gated ion channel (GLIC)	**cation channel**
**4LP8**	Inward rectifier potassium channel Kirbac3.1	**K+ channel**
**4LTO**	Bacterial sodium channel (in high calcium)	**Na+ channel**
**4MS2**	Voltage-Gated Calcium Channel (CaV) created by mutation of the NaVAb channel	**Ca2+ channel**
**4NEF**	Aquaporin-2 AQP2	**water channel**
**4NPP**	Prokaryotic pentameric ligand-gated ion channel (GLIC)	**cation channel**
**4NTW**	Acid-sensing ion channel 1	**Na+ channel**
**4NYK**	Acid-Sensing Ion Channel 1 ASIC1	**Na+ channel**
**4OXS**	Prokaryotic sodium channel from Magnetococcus marinus (NavMs)	**Na+ channel**
**4PE5**	Heterotetrameric GluN1-GluN2B NMDA receptor ion channel	**Ca2+ channel**
**4PGU**	YetJ from Bacillus Subtilis at pH 7	**Ca2+ channel**
**4RDQ**	Bestrophin-1 (BEST1) Ca2+-activated Cl- channel	**chloride channel**
**4TNV**	Glutamate-gated chloride channel alpha (GluCl) (non-conducting state)	**chloride channel**
**4UUJ**	KcsA Potassium channel, H+ gated	**K+ channel**

PDB ID	Name	Selectivity
**4WD7**	Bestrophin homolog Ca2+-activated Cl- channel	**Na+ channel**
**4WFE**	Two-Pore Domain Potassium Channel K2P4.1 (TRAAK)	**K+ channel**

REFERENCES

(1) *Handbook of Chemistry and Physics*; 87th ed.; Lide, D. R., Ed.; CRC Press: Boca Raton, 2006.

(2) Friedman, H. L.; Krishnan, C. V. In *Water: A comprehensive treatise*; Franks, F., Ed.; Plenum Press: New York, 1973; Vol. 3, p 1.

(3) Ben-Naim, A.; Marcus, Y. *J. Chem. Phys.* **1984**, *81*, 2016.

(4) Chambers, C. C.; Hawkins, G. D.; Cramer, C. J.; Truhlar, D. G. *J. Phys. Chem.* **1996**, *100*, 16385.

(5) Wolfenden, R. *Biochemistry* **1978**, *17*, 201.

(6) Lim, C.; Bashford, D.; Karplus, M. *J. Phys. Chem.* **1991**, *95*, 5610.

(7) Ozutsumi, K.; Ishigiro, S. *Bull. Chem. Soc. Jpn* **1992**, *65*, 1173.

(8) Smith, R. M.; Martell, A. E. *Sci. Total Environ.* **1987**, *64*, 125.

(9) Dudev, T.; Lim, C. *Phys. Chem. Chem. Phys.* **2012**, *14*, 12451.

(10) Dudev, T.; Lim, C. *Sci. Rep.* **2015**, *5*, 7864.

(11) Kellenberger, S.; Hoffmann-Pochon, N.; Gautschi, I.; Schneeberger, E.; Schild, L. *J. Gen. Physiol.* **1999**, *114*, 13.

(12) Dudev, T.; Lim, C. *J. Am. Chem. Soc.* **2010**, *132*, 2321.

(13) Sun, Y. M.; Favre, I.; Schild, L.; Moczydlowski, E. *J. Gen. Physiol.* **1997**, *118*, 693.

(14) Baconguis, I.; Bohlen, C. J.; Goehring, A.; Julius, D.; Gouaux, E. *Cell* **2014**, *156*, 717.

(15) Payandeh, J.; Scheuer, T.; Zheng, N.; Catterall, W. A. *Nature* **2011**, *475*, 353.
